# What is the perceived relationship between boredom and substance use among persons who are unhoused?

**DOI:** 10.1371/journal.pmen.0000354

**Published:** 2026-02-06

**Authors:** Cory Herzog-Fequet, Rebecca Gewurtz, Noah Hansen, Halley Read, Carrie Marshall

**Affiliations:** 1 Social Justice in Mental Health Research Lab, Faculty of Health Sciences, University of Western Ontario, London, Ontario, Canada; 2 School of Rehabilitation Science, McMaster University, Hamilton, Ontario, Canada; 3 School of Occupational Therapy, Arkansas Colleges of Health Education, Fort Smith, Arkansas, United States of America; 4 School of Occupational Therapy, Western Oregon University, Monmouth, Oregon, United States of America; 5 Social Justice in Mental Health Research Lab, Faculty of Health Sciences, University of Western Ontario, London, Ontario, Canada; University of Milano–Bicocca: Universita degli Studi di Milano-Bicocca, ITALY

## Abstract

While there is much research suggesting that there is an association between the experience of boredom and the use of substances, the mechanisms which govern this relationship are largely unknown. This study was employed to generate an enhanced understanding of the relationship between boredom and substance use among persons who are unhoused living in high-income nations. A secondary analysis of 18 qualitative interviews from persons experiencing homelessness was conducted utilizing reflexive thematic analysis. Sen’s Capabilities Approach was used as a framework to guide this abductive analysis. The central essence of participant interviews was: “*idle hands here are the devil’s playground*”. Within this essence, two themes were generated: 1) the contextual conditions influencing substance use; and 2) substances as an escape from boredom The experience of boredom was identified by participants as a factor that increased the propensity to engage in substance use. Numerous mechanisms influencing this relationship were identified. This study illustrates the importance of providing unhoused persons with opportunities to engage in meaningful activities to alleviate the boredom that factors so heavily into their lives. The findings of this research emphasize the importance of addressing boredom among persons experiencing homelessness and may be used to better inform policy, practice, and future research endeavors.

## Introduction

The experience of boredom among persons who are unhoused may seem trivial when compared to other issues this population experiences, such as higher rates of morbidity and mortality than the general population [1], however, has been identified as a common and pervasive experience for persons who are unhoused [2,3]. Researchers have long associated boredom with the use of substances [4]. Only a handful of studies have explored this issue among persons who are unhoused [3,5,6], and none are known to focus on this phenomenon as central construct of interest.

### What is boredom?

Boredom has long been a topic of discussion in philosophy [7]. Recently, the topic of boredom has seen a proliferation of research in a wide variety of academic disciplines [7]. Despite the long history of study and recent proliferation of research, the concept of boredom does not have a universally accepted definition [8]. It is important to note, however, that boredom is conceptually distinct from related concepts such as depression or apathy [9,10] and the experience of boredom entails unique physiological and neurological responses in individuals [11]. Boredom occurs when there is a mismatch between the requirements of the task at hand and individual capabilities, preferences, and energy levels [7]. The experience of boredom often involves a perceived slowness in the passage of time [12] or a lack of meaning that results in a strong desire to perform alternative activities [13]. The evolutionary purpose of boredom is to signal that the current task or state of being is unvaluable, thereby encouraging engagement in alternate pursuits [7]. This signal in turn triggers an undirected response whereby individuals search for *anything* that may yield better functioning, or better matches capabilities, preferences, and energy levels [7].

Individuals may manage boredom using coping strategies that positively or negatively impact psychosocial wellbeing. Experiencing boredom can be considered adaptive in that it promotes creativity and curiosity [14] and facilitates psychological growth [15]. It is noteworthy to mention however, that the feeling of boredom itself is aversive [16], and that this aversion increases proportionately over time [7]. When experienced as a chronic condition boredom can induce feelings of suffering and impede personal development [15]. Prolonged boredom can therefore be considered deleterious for the psychological health of individuals [2]. Similarly, extended durations of boredom have been found to contribute to engagement in deleterious behaviours that can be construed as a threat to individual wellbeing [17].

One such coping behaviour related to boredom that may have the propensity to negatively impact psychosocial wellbeing is that of substance use. This is especially true among persons experiencing homelessness with previous research suggesting that individuals utilize substances when there is a lack of meaningful activities available within their environments [18]. The high levels of boredom can be attributed, at least in part, to the environment in which persons who are unhoused often find themselves which are often highly structured and institutional in nature, leading to a lack of autonomy and freedom for persons who are unhoused [6,19]. Indeed, service providers may actively be contributing to the experience of boredom among persons who are unhoused by restricting meaningful activities under the premise that high levels of boredom may increase the motivation of service users to find housing and cease or limit shelter usage [18]. This is concerning given that researchers interested in homelessness have found that extended durations of boredom contribute to the initiation or maintenance of substance use within this population [19–21]. Similarly, existing housing support models do not adequately address the issue of substance use among persons experiencing homelessness [22] and community-based interventions often entail substantial barriers that prevent persons who are unhoused from accessing or adhering to treatment regimens [23]. The chronic experience of boredom and the inadequacy of existing support models to address substance use may help to explain the high rates of substance use within this population, estimated at 53% [24].

When confronted with widespread social marginalization, unhoused individuals often gather in the same geographic locations and socialize with others facing similar levels of social exclusion, which can lead to similar patterns of substance use [19,25]. Alternative ways to cope with environmental boredom are often too costly and out of reach for those who are unhoused resulting in a culture of reciprocity centered predominantly on the shared acquisition and use of mind-altering substances [26]. This reliance on substances to mitigate the negative psychosocial effects of boredom results in chemical dependencies and detrimentally impacts daily functioning [18,26] arguably prolonging the state of homelessness [27]. Interestingly, studies suggest that the provision of meaningful activities may play an important role in the reduction of both boredom and substance use among persons experiencing homelessness [28,29]. It is noteworthy that there are no known studies that purposefully seek to elucidate the mechanisms governing the relationship between substance use and boredom within this demographic. There is a clear need to further examine the nature of this relationship to better inform policy and practice that adequately addresses the importance of boredom among persons experiencing homelessness.

### The Capabilities Approach

The Capabilities Approach, as originally conceptualized by Sen [30] and furthered by Nussbaum [31], was used to guide the interpretation and analysis of this study. This approach falls within a social justice framework that recognizes the inequities in the allocation of material resources throughout society [32]. Unlike other social justice theories, the Capabilities Approach shifts the focus from the distribution of resources to prioritizing individual freedom and the varying capacities people have to convert resources into their own concept of a fulfilling life [33]. In this sense, capabilities refer to the freedoms that people have or don’t have to be able to live the life they want [34]. Sen [33] and Nussbaum [31] contend that the central concern of the capabilities approach is what individuals can achieve with their available opportunities and resources. Central to this theory is the role of “functionings,” or what an individual is able “to do and to be” (p.18) given the capabilities (opportunities and resources) available to them [31]. Capabilities may therefore be conceptualized as the raw resources or opportunities that an individual has available to them, whereas the functionings are what that person chooses to do with these capabilities. An important aspect of this theory is therefore the freedom of choice [32]. This approach emphasizes assessing human development through the lens of personal autonomy and freedom [35]. Rather than solely addressing health disparities, the Capabilities Approach takes a broader view that encompasses inequities in social determinants of health [32].

Nussbaum’s [31] identifies ten capabilities that are necessary for a dignified and fulfilling human life. These capabilities represent essential areas where individuals should have the opportunity to develop and exercise their potential. The first capability is that of *life*, whereby individuals should have the ability to live an average lifespan relative to other individuals in the same society. *Bodily health* refers to the capability to enjoy good health, access to necessary medical care, and the opportunity to lead a life free from severe physical ailments. *Bodily integrity* refers to being free from physical harm and violence as well as the autonomy to make choices about one’s own body and personal safety. *Senses, imagination, and thoughts* refers to the ability to use one’s senses and imagination, to think critically and creatively, and to engage in intellectual pursuits. The capability of *emotions* is related to the ability to experience and express emotions such as love, grief, and joy, and to have the support needed to manage emotional well-being. *Practical reason* is the capacity to form and pursue a conception of a good life, make informed decisions, and engage in reflective thinking about one’s values and life goals. *Affiliation* refers to the opportunities to form social connections and relationships, participate in social interactions, and be treated with respect and equality. The capability of *other species* refers to the ability to live in harmony with other species and the environment, acknowledging the interconnectedness of all life forms and the need for environmental sustainability. *Play* refers to opportunities to engage in recreational activities and enjoy leisure, which is vital for personal development and well-being. The final capability is *control over one’s environment* which is the ability to participate effectively in political and economic activities, have access to meaningful work, and influence decisions that affect one’s life and community.

The Capabilities Approach was chosen as the guiding framework for this study due to its comprehensive approach to social justice which entails addressing the disparities in the social determinants of health and emphasizing personal freedom of choice. This perspective is particularly valuable for understanding contexts of severe inequality where individuals lack essential physical, social, and personal resources needed for autonomy [35]. This inequality is apparent among persons who are unhoused as the state of homelessness withholds the necessary resources that individuals require to have autonomy in their lives [34,35]. Homelessness is a form of extreme social exclusion, with individuals experiencing a range of unmet needs [5]. These unmet needs can be understood as an inability to access the essential capabilities as described by Nussbaum [31]. Social institutions, within this framework, should establish the conditions that enable people to pursue diverse notions of a good life [32]. Applying the Capabilities Approach therefore provides a valuable lens for evaluating current services and advocating for systemic improvements [36].

## Materials and methods

The presence of profound boredom and how this experience influences or is influenced by substance use among persons who are unhoused is a topic worthy of further exploration. While there are studies that allude to the relationship between boredom and substance use among the homeless population [3,18,20,21], none have focused on this association as the primary research question. This study aims to address this literary dearth by asking the question: what is the perceived relationship between boredom and substance use among persons who are unhoused?

### Methodology

To answer this question, we conducted a secondary analysis of data from a mixed-methods study exploring boredom during and following homelessness [5]. For the current study, only qualitative interviews with individuals who were unhoused at the time of data collection in the parent study were analyzed.

We analyzed the data using reflexive thematic analysis [37]. Braun and Clarke indicate that when using reflexive thematic analysis, researchers must explicitly identify their ontological and epistemological positioning for their study [37]. In this study, an idealist ontology and an interpretivist epistemology were utilized. Interpretivist approaches acknowledge that there are multiple realities determined by the perceptions of individuals and shaped through social interaction [38]. These approaches typically employ qualitative data and recognize that truth and knowledge are contingent upon individuals’ interpretations of reality across various histories and cultures [38].

### Ethics statement

Ethics approval was granted by Western University to the principal investigator of the parent study (C. Marshall) who is a co-author on the current study. Participants were recruited over a one-year period from June 19, 2019, to March 13, 2020 [5]. All participants in the parent study read a letter of information before signing a form indicating their consent to participate in interviews [5].

### Procedure

Qualitative interviews were conducted in the parent study after quantitative interviews were completed. Participants received $40 in compensation for partaking in mixed interviews. Participants engaged in semi-structured interviews designed to explore the experience of boredom during and after homelessness, although only those interviews involving participants who were unhoused were used to inform this secondary analysis. Purposive sampling was utilized in the parent study to ensure a range of participant ages, genders, housing status, and geographic locations. Interviews were digitally recorded and transcribed verbatim.

### Recruitment

Participants were recruited from shelters, drop-in centres, housing case management, and housing programs in three mid-sized cities located in Ontario, Canada (Kingston, London, and Hamilton). Recruitment included a combination of purposive, snowball, and convenience sampling. In the parent study, the researchers gathered qualitative and quantitative data from individuals who were either unhoused for at least one month in the past year or housed for less than three years following homelessness [5]. In this secondary analysis, we have used a subset of the available data, specifically the data generated from qualitative interviews of persons who were currently unhoused. Participants chose a pseudonym to ensure confidentiality.

### Inclusion and exclusion criteria

Inclusion criteria for the data used in this secondary analysis consists of individuals over the age of 18 years who were unhoused for at least one month in their current episode of homelessness. Individuals who were recruited had a history of being unsheltered or emergency sheltered as defined by the Canadian Observation on Homelessness [39].

### Analysis

Data was analyzed by the primary author of this paper, a postgraduate student at the time, and reviewed by coauthors of this paper. The primary author of this paper has completed postgraduate research methods courses and performed the analysis with guidance from his thesis supervisor, Dr. Carrie Marshall (coauthor). All contributing authors apart from the primary author have a long list of previous publications, including qualitative research studies.

Data analysis was completed using reflexive thematic analysis [37]. Thematic analysis is a method for analyzing qualitative data [40] that attempts to capture an overarching “essence” of the data [41]. Thematic analysis consists of six steps [41]: 1) become familiar with the data through reading and re-reading transcripts without the generation or application of codes; 2) generation of initial codes; 3) analysis of initial codes for the purpose of theme generation; 4) reviewing the initial themes looking for further refinement and enhancing coherence; 5) identify the essence of the themes and name the themes accordingly; and 6) final analysis and writing the findings. As recommended by Thompson [40], three rounds of coding were conducted after the initial readthrough. Coding was completed via the use of Dedoose, a cloud-based qualitative data management software used to organize the corpus data [42].

It should also be noted that coding was done abductively. Abductive analysis blends inductive and deductive approaches and allows for the application of a theoretical lens to guide data analysis [43]. In contrast, deductive approaches attempt to generate a singular guaranteed truth [43], whereas abductive analysis offers speculative explanations to a specific phenomenon [44]. Rather than engaging in the analysis of data without any priori assumptions, abductive analysis engages the data with a theoretical understanding that sets parameters on what is sought within the data [40]. In this instance, this theoretical understanding is provided via the incorporation of the Capabilities Approach.

#### Trustworthiness.

Trustworthiness was achieved utilizing criteria established by Lincoln & Guba [45]. The first criterion is that of credibility. Credibility was met via prolonged engagement with the subject population. All members of the research team have extensive experience working with persons who are unhoused, either in practice or research capacities. Peer debriefing strategies were utilized with frequent discussions occurring as needed with other academics in the Social Justice and Mental Health Lab at Western University as well as among the co-authors. Negative case analysis was also conducted, which acknowledged any instances where participant voices were not consistent with the overarching theme [45]. To ensure transferability, thick descriptions of the data were incorporated into the analysis via the use of detailed descriptions and quotes. Dependability was ensured via frequent review by research members and engaging in reflexivity (see below). Confirmability was facilitated using qualitative data management software [42], maintenance of an audit trail, and ongoing discussions among the research team.

#### Reflexivity.

The primary author of this paper has approximately seven years of experience in providing health and social care services to persons experiencing homelessness, while the rest of the research team collectively has decades of experience working in practice and research with persons who are unhoused. Similarly, the primary author has also experienced episodic homelessness as a youth, and therefore has some firsthand experience with the stigma and social isolation associated with this state of living, albeit for relatively short durations. Rather than being detrimental, this collective experience on the part of the research team is believed to have contributed to the analysis of participant narratives with a greater level of understanding and depth.

## Results

### Participants

A total of n = 18 participants were included in this secondary analysis. Of these, n = 9 (50%) identified as men, and n = 9 (50%) identified as women. The median age of participants was 38 (IQR = 10; range = 38). Participants represented three cities in Ontario, Canada including London (n = 7; 39%), Hamilton (n = 5; 28%), and Kingston (n = 6; 33%). See Table 1 for demographic information.

**Table 1:** Participant demographic characteristics.

n=(18)	
Demographic characteristics	n (%)
Gender	
Male	9 (50)
Female	9 (50)
Other	0 (0)
Age	Mdn = 38
	Range 18–56
	IQR = 10
Race/ethnicity	
Caucasian/white	14 (77.8)
African Canadian/black	1 (5.6)
Indigenous	2 (11.1)
Mixed ancestry	1 (5.6)
Months unhoused in the past year	Mdn = 9
	Range 1–12
	IQR 7.5
Age at first episode of homelessness	Mdn = 29
	Range 15–55
	IQR 15.75

### Qualitative findings

The overall essence that we generated in our analysis of interviews was: “*idle hands here are the devil’s playground*”. Throughout the interviews, many participants discussed meaningful activities and how they relate to substance use. More specifically, participant responses seemed to suggest that the ability to engage in activities that were productive, meaningful, or enjoyable was something of a protective factor regarding the engagement in negatively conceptualized activities such as substance use. Boredom caused by a lack of activity was construed as something that needed to be guarded against to avoid eliciting the drive to use substances. This perception can be summed up in the following quote: “*So, if you’re not catching that boredom, that dangerous boredom, and doing something productive with it, you’re only allowing yourself to substance abuse.*” [Susan]. The lack of productive activities available within the environment was seen as a risk for substance use. On a related note, prosocial activities were often recommended by participants, but these activities were often unattainable given environmental and economic constraints: “*Like there’s no arts and crafts room, you know, nothing like that, and that would likely save them a lot of problems… how does that old phrase go? Umm idle hands here are the devil’s playground.”* [Shawn]. Others lamented the loss of activities that were once available: “*Like when we had movie nights, a lot of people went to movie night, so that’s like 2 hours where no one is doing drugs right?*” [Taylor]. These activities, despite being identified as meaningful by participants, were not available in the shelter environment or in the community. With such constraints negatively impacting their ability to engage in anything the participants identified as meaningful, many resorted to the use of substances or languished in a state of prolonged boredom.

Throughout interviews, participants overwhelmingly discussed how their constrained abilities to engage in meaningful activities resulted in boredom, and how many coped through the use of substances. This essence was captured in two overarching themes: 1) The context of boredom and 2) Substances as an escape from boredom.

***Theme 1: The context of boredom.*** Most participants discussed the environmental conditions that directly influence the propensity to engage in substance use. These discussions occurred within the backdrop of constrained autonomy resulting from limited physical or geographic mobility due to reliance on emergency shelter supports and accompanying policies. Participants spoke about how their reliance on these supports dictated their time use and influenced their substance use habits: “*If let’s say in between here and there, so this place closes at 8:30, that place doesn’t open until 10. A lot of people go in between and use*” [Denver].

Participants also made specific references to the limited ability they felt they had to engage in meaningful activities that were unavailable within their respective environments:

*I find people that just stay here, they don’t really do anything, they’re always, like I said when I come back, I always see people on their phones, or got a headset on, or they’re on the computer, smoking, they sit out there and smoke cigarette after cigarette.* [Jimmy]

This constrained autonomy resulting from both limited mobility that was dictated by the services participants relied upon to meet their basic needs and the overall lack of meaningful activities within these environments led to a unique subculture centred largely around the collective consumption of substances: “*Yeah, because we, we had nowhere to be. So, we ended up in bad places (sniffles), and like along with those places comes a certain type of people (sniffles). And, that’s where like drugs and stuff come into play.*” [Denver].

All these factors contribute to the use of substances within this population. This theme was expressed through the following sub-themes: 1) “*It’s in your face*” and 2) *How boredom and substance use facilitate interpersonal relations*.

*“It’s in your face”*. Many of the interview participants spoke about the ease of access regarding substance use within their respective environments and how this negatively influenced the ability to abstain from substances in the face of boredom. As one participant described regarding the prevalence of substances within the various shelters that were accessible to them: “*Umm, it’s just 3 men’s shelters. The other two are very uh drugs, like it’s in your face, I guess you could say.*” [Hamilton]. Participants described this experience both within the context of the shelter environment itself as well as within the community generally. One participant attempted to quantify the prevalence of substance use among his peers: “*Like we’re not even talking in 60-70%, we’re talking 80-90% of people.*” [Shawn]. Another participant found it easier to provide a numeric value for the total number of individuals they know who do not use substances: “*There is honestly, let’s just say the women that come in here that are homeless that I can think of that are like in my life. I only know of two of them who don’t use drugs.”* [Denver]. Some of the participants accepted this aspect of their social predicament and engaged in substance use with their peers: “*When in China, you eat rice you know. I don’t know how else to put it, you kind of go with the flow… It’s hard not to get into all the stuff.*” [Shawn]. For those that did not wish to do so however, strategies to avoid substances often revolved around avoiding specific locations or individuals. Some participants conceptualized leaving the confines of the shelter itself as a risk factor for substance use: “*If you’re out there* [in front of the shelter] *and hanging out with all of them, you’re either going to get stoned or drunk. I guarantee it, it’s one of those things, it’s copious amounts of drugs, lots of alcohol out there.*” [Shawn]. These avoidant strategies resulted in increased social isolation whereby individuals purposefully chose to disengage from their peers or remain sequestered within the institutionalized shelter environment arguably contributing to the experience of prolonged boredom. It is telling to note that the perceived options these participants had available to them were constrained, with the available choices being to engage in substance use with peers, sleep, or languish in an uninterrupted state of boredom:

*For me, if I have nothing. Like if, sleeping my days away it’s a good thing in a sense, if I was bored I would be…probably going and hanging out with people I shouldn’t be, or* [to] *houses - the only place I know now. The only people I know…around things I shouldn’t be around. A lot* [of] *people that use…I think I’d end up going down that road.* [Summer]

As this excerpt demonstrates, the primary strategy to avoid engaging in substance use with peers was to sleep, thereby limiting the ability to engage in meaningful activities, develop supportive or prosocial peer networks, and further constricting their autonomy to move freely within the community. The prevalence of substance use within this population serves as a barrier to abstinence, with participants either engaging in avoidant strategies that perpetuated the experience of boredom or choosing to “*go with the flow*” [Shawn] and similarly engaging in the use of substances with their peers in the absence of alternative methods of meaningful interpersonal interactions.

*How boredom and substance use facilitate interpersonal relations.* Some participants referred to subcultures within the unhoused population that related to boredom and its associations with substance use. Specifically, some participants discussed the sense of community they felt when utilizing substances with other individuals similarly experiencing prolonged boredom while unhoused. As one participant described when speaking about substances:

*They give me something to, as shitty as it is, it gives me something to do, I mean, I hate to say this but I made a lot friends doing bad things, like I’ve met a lot of people that really related to me, who connected with me through everything awful I’ve done, like as much as drugs are awful, I’ve met some really interesting people*. [Taylor]

This excerpt demonstrates that while the reliance on substances as a means of social engagement is conceptualized as negative, it nonetheless plays a role in facilitating social relationships. A similar interpretation was offered by Summer when speaking about the use of substances among her peers: “*So, if I was bored, and not able to sleep, I would probably end up partaking and hanging around people of such kind*”. These discussions regarding a community of individuals using substances occurred within a larger discussion of social marginalization and exclusion from society generally. Boredom arising from exclusion from meaningful activities due to extreme social and economic exclusion led to the collective use of substances among individuals who are unhoused. This subculture is recognized as separate from the general public by at least some of the participants. Rather than concerning oneself with the inability to achieve normative status symbols, such as a formal education, this participant engages in substance use with his peers to not “*worry about it at all*”: “*For the most part crystal [methamphetamine] is cheaper to committing to what society wants, which is you know like uh having paid tuition … you know I don’t have to worry about it at all.*” [Street Jesus]. Persons who are unhoused do not have the same ability to engage in meaningful activities as the general public, and at least some of them develop and maintain social networks that revolve around the use of substances while eschewing more normative (and largely unattainable) aspirations and ways of life.

***Theme 2: Substances as an escape from boredom.*** This theme addresses the specific mechanisms which may govern the relationship between boredom and substance use among persons who are unhoused. More specifically, participants described substance use as a direct consequence of experiencing boredom. Boredom was perceived as an antecedent to the engagement in substance use, with substance use being conceptualized as a means of coping with this experience. This boredom and cooccurring substance use materialize in an environment construed as largely devoid of purposeful activities. As explained by one client: “*A lot of people just don’t do anything, they’ll take, they’ll take boredom and then replace it with drinking, booze, drinking, drugs…”* [Jimmy]. In their respective environments, persons who are unhoused are unwilling or unable to engage in meaningful activities, resulting in the experience of boredom. This experience, particularly in a prolonged state, is seen as unpleasant and often results in the use of substances. This theme was associated with four sub-themes: 1) *Boredom as a negative cognitive or emotional state managed with substances*; 2) *Substance use provides stimulation when bored*; 3) *Substance use kills meaningless time;* and 4) *Substance use as the most affordable way to escape boredom.*

*Boredom as a negative cognitive or emotional state managed with substances*. Participants identified that boredom was an unpleasant experience resolved or mitigated via the use of licit or illicit substances. As such, boredom was typically equated with a similar emotive state, such as depression, or was seen as a cognitive state that contributed to the relieving of past traumas, grief, or similar negative recollections. As one participant described, boredom results in “*that stinking thinking*” [Hamilton] because the mind is allowed to ruminate upon unpleasant emotions and cognitions. Susan elaborated on a similar conceptualization when asked about substance use in her environment: “*They’re masking their feelings, or self uh what’s the word I’m looking for, self-medicating through their boredom you know. Because otherwise they’d have to think about it*.” Rather than thinking about boredom and the negative emotions or cognitions that coincide with this emotive state, persons who are unhoused often choose to utilize substances as a means of coping with this experience. Some participants described the use of substances to help forget or distract themselves from their anxiety, depression, or other negative emotional states that boredom was said to exacerbate. Others similarly spoke about how the experience of boredom leads to thoughts of loss, and how substance use mitigates these thoughts: “*Like the type of boredom I was experiencing was because of like loss (sniffles)… it makes you feel like shitty about yourself (sniffles), so that definitely leads to drug use.”* [Denver]. There was no singular cause of loss identified, but specific examples of loss provided by participants include the loss of children, family ties, physical capability, occupation, or a previous (housed) lifestyle. The lack of meaningful activities available in the participant’s environments appears to contribute to ruminations about their experiences of loss, including the loss of meaningful individuals, activities, or abilities. This rumination then contributes to negative feelings or cognitions, which are in turn managed with the use of substances. Regardless of the whether participants describe experiencing a negative emotive or cognitive state, the implication is the same: substance use helps mitigate the negative effects of boredom.

*Substance use provides stimulation when bored*. Another explanation for the relationship between boredom and substance use among unhoused participants includes the provision of stimulation otherwise unavailable in the environment. This explanation occurred within the context of a lack of interesting activities to otherwise occupy the mind. As one participant described, substances were used to break up the “*monotony*” [Shawn] of the day. Others described substances as bringing out “*alertness*” [Speedy] or stimulating “*brain activity*” [Speedy]. All these interpretations of boredom allude to something of a mental numbness or general malaise due to a lack of meaningful activities available within their environment. Rather than succumb to this experience, some participants utilized substances to artificially produce mental stimulation. Substances specifically referenced within this interpretation include cannabis, alcohol, or, as one participant described, the individual’s “*favourite drug*” [Shawn]. It is interesting to note that the alternative to using substances as a source of stimulation was not the engagement in a meaningful activity, but rather going to sleep for extended durations. When asked about alternative activities, Summer replied: “*Umm, I don’t participate in many activities of any kind. Right now, I tend to just sleep my days away*.” Denver had a similar perception when discussing the activities of her peers: “*So you’re going there* [to the shelter] *and just go to sleep, or whatever, some stay up all night. You can be under the influence* [of substances] *there*”. Participants felt unable to engage in stimulating activities while unhoused and were therefore left with only two constrained choices: sleep or substance use. Both of these choices can in turn be conceptualized as a means of escaping the unpleasant experience of boredom, an escape not otherwise accessible via other means. With a lack of agency to be able to engage in alternative meaningful or stimulating activities, some of these participants chose to use substances as the preferred means of coping with these constrictive environmental conditions.

*Substance use kills meaningless time.* Substance use was also utilized by some participants because it was perceived as filling time or making the day go quicker. In this conceptualization, boredom was construed as a slowed passage of time devoid of meaningful activities. As one participant stated: “*The beer and weed actually speeds up the day.*” [Speedy]. Similarly, other participants described the use of substances as “*something to do*” [London], thereby implying that there was very little else available to do within their environments. Interestingly, some of these same participants spoke of abstinence from substance use as filling their day with other (nonsubstance related) activities. This dualistic interpretation would seem to imply that when one is able to find activities to fill their time it leads to a reduction or cessation of substance use, but when activities are unavailable substance use can be substituted as a means of occupying leisure time and mitigating the effects of boredom. Boredom can be conceptualized as a slowed passage of time due to a dearth of meaningful activities available to unhoused participants, itself caused by economic marginalization, social exclusion, or environmental constraints, with substance use acting as a means of filling up these large swaths of meaningless time.

*Substance use as the most affordable way to escape boredom*. The reason for persons who are unhoused utilizing substances as a means of coping with boredom may lay in the socioeconomic marginalization that characterizes this population. Throughout the interviews participants specifically referenced the use of substances to cope with boredom due to financial constraints: “*And then you go for coffee and smoke cigarettes, that’s it. You can’t afford anything else.*” [Apple]. Participants discussed how alternative activities which may have been meaningful or otherwise occupy time were unaffordable, but the use of substances was not. Some talked about how homelessness involved teaching themselves how to fill their time without money: “*I used cannabis too right? As a coping mechanism so, you just you kind of start off by teaching yourself how to fill your time without money.*” [Susan]. Others referenced specific activities as too expensive and related them to the use of substances, which were conceptualized as more affordable. One participant described substance use as an escape from the detrimental effects of boredom:

*Well basically you just, you roll yourself into getting as high as possible, so you can stare at the sky, at the stars and pretend you’re flying through them or something because life sucks (chuckles), so you need an escape. We can’t take vacations, we can’t take a day off and go do something, or have the money, you can’t even go to like for example a zoo or something like that, because I really don’t have the money.* [Shawn]

Due to economic constraints necessarily limiting their autonomy to engage in other meaningful activities, persons who are unhoused may resort to “*getting as high as possible*” as a means of escaping their situational predicament. Implied within this articulation is the notion that, were other activities accessible, this reliance on substance use as an escape from the unpleasant experience of boredom may not be required. Statements such as these highlight the economic scarcity that underpin the high prevalence of substance use within this population. People experiencing homelessness have few practical ways to alleviate the profound boredom that often comes with their situation, thereby resorting to substance use as a means of coping with this unpleasant experience.

## Discussion

This study was conducted to further explore the perceived relationship between boredom and substance use among unhoused persons in high-income nations. This is the only known study which focuses on the relationship between boredom and substance use among persons who are unhoused. Our findings build upon existing research and support the assertation that boredom contributes to the use of substances within this population [19–21]. This study also contributes to a growing body of literature pertaining to the importance of meaningful activities in the lives of individuals experiencing homelessness [21,46,47] and can be used to inform approaches that reduce barriers to access. The Capabilities Approach underscores that the ability to flourish requires the empowerment of individuals to engage in meaningful activities that reflect their values and aspirations; opportunities which were largely denied to the participants in this study.

The capability of *life* refers to the ability to live an average lifespan and the capability of *bodily health* refers to the ability to enjoy good health and avoid otherwise avoidable health conditions that may negatively impact life [31]. Participants spoke at length about how contextual factors contribute to a dearth of meaningful activities resulting in both boredom and the use of substances. The prevalence of substance use among persons experiencing homelessness serves as a contributing factor in the use of substances, particularly when the alternative is incessant boredom, which may in turn carry important health implications. The fact that the environmental conditions themselves may contribute to the use of substances is concerning given that persons who are unhoused experience high rates of substance use which may in turn contribute to the high prevalence of mental illness [48] and morbidity found within this demographic [1]. Furthermore, engagement in substance use has been identified as a contributing factor of ongoing homelessness [49]. As such, targeting the high prevalence of substance use may serve as a means of reducing the duration of homelessness [49], which itself may be conceptualized as a threat to both psychosocial and physical wellbeing given the health disparities associated with homelessness. One possible means of addressing this substance use, as elucidated by the participants themselves, is via the provision of meaningful activities. Interviews highlighted the importance that individuals gave to the ability to engage in meaningful activities while simultaneously lamenting their inability to do so within their current environment. The ability to engage in alternative activities was thought to reduce the experience of boredom, thereby mitigating the perceived need to utilize substances as a means of coping with this unpleasant experience. Mitigating boredom in this way may be an important avenue of exploration as a means of reducing substance use and improving the health outcomes of individuals experiencing homelessness.

Nussbaum’s [31] capability of *bodily integrity* refers to the freedom to practice individual autonomy over one’s movement and protection from trauma. Throughout the interviews, participants discussed an inability to exercise autonomy over movements throughout the community, which contributed to the experience of boredom. Previous research has shown that persons experiencing homelessness often have their use of time dictated by basic needs programs and shelters [19,50], a finding supported by this study. These constraints on bodily integrity resulted in a surplus of unfulfilled time with participants often resorting to the use of substances to “fill time”. Additionally, the experience of boredom among participants often led to ruminations about past trauma. This reliving of trauma was perceived as unpleasant with engagement in substance use seen as a means of mitigating these unpleasant cognitions. While this study did not delve specifically into the experience of trauma itself, participants’ statements support the notion that persons who experience homelessness are frequently exposed to trauma [5,51]*.*

Participants also discussed how they share their environment with people who were not necessarily of their choosing due to the nature of emergency housing services placing persons experiencing homelessness together in one limited physical location. This approach often resulted in feelings of social isolation and loneliness despite the presence of other individuals in close proximity. Increasing the ability for persons who are unhoused to move freely and without constraint may help to minimize the level of pervasive boredom experienced, which may in turn improve psychosocial wellbeing by reducing the reliving of trauma, loneliness, and coinciding substance use.

The capability of *senses, imagination, and thought* pertains to the ability to exercise reasons and intellect, typically through education, and opportunities to engage in activities of interest while the capability of *play* relates to the ability to engage in recreation [31]. These capabilities were largely denied to participants, contributing to the experience of boredom and use of substances. Education, at least in the formal sense, was conceptualized as unrealistic by participants due to economic constraints. However, as education was not a primary focus of the original interviews, these observations were raised by only a portion of participants and should be interpreted as illustrative rather than representative. The ability to engage in meaningful activities was also denied to participants, both within the shelter and the larger community. Recreational opportunities were unavailable within shelters and were seen as unattainable in the community. The economic deprivation that participants experience were seen as barriers to engaging in meaningful activities within the community. While participants described a desire to engage in activities that do not involve the use of substances, these opportunities were seen as too expensive when compared to the relative ease and affordability of accessing and using substances. This conceptualization suggests that the socioeconomic marginalization that afflicts persons who are unhoused is inseparable from the corresponding boredom and accompanying substance use this experience necessarily entails. Interestingly, none of the participants in this study expressed having the ability for employment. It should be noted however that the interviewers did not specifically inquire as to whether participants were employed. As such, the absence of direct questions about employment represents an important limitation, as it restricts the ability to determine how occupational opportunities—or their absence—may have contributed to participants’ experiences of boredom and substance use. It therefore remains unclear whether participants lacked the specific socioemotional skills necessary to identify and follow through with the procurement of meaningful activities, in addition to the socioeconomic barriers that were identified throughout participant interviews. Research has demonstrated that persons experiencing homelessness often face limited opportunities for employment due to time spent acquiring basic necessities (such as food or shelter), lower educational attainment, and restrictions imposed by income support programs [52]. This lack of employment may contribute to the ongoing poverty that participants described as a barrier to participating in meaningful activities. This unaddressed poverty is important, as poverty is a major contributor to ongoing homelessness [53] and prevents engagement in meaningful social or recreational activities [52]. Faced with an inability to manage boredom through meaningful activity, participants often resorted to the use of substances.

The capability of *emotions* refers to the opportunities necessary to form attachments to other humans and express emotions while the capability of *affiliation* pertains to the ability to live and interact with others while being viewed as an equal [31]. Participants described a multitude of barriers to achieving these capabilities, which contributed to the experience of boredom and the use of substances as a result. Throughout participant interviews, numerous expressions of loss and grief were made, often related to former friends and family. This social isolation was exacerbated by the experience of chronic boredom. Many participants spoke about how their lack of financial resources contributed to social exclusion as they felt they were unable to readily engage in interpersonal relationships with individuals who have greater socioeconomic means than themselves. In the face of this social exclusion, some participants formed relationships centred around the use of mind-altering substances, a finding previously reported among persons who are unhoused [26]. Substance use was described by participants as playing a substantial role in the creation and maintenance of interpersonal relationships. The alternative to conforming to this subculture where interpersonal relationships are established or maintained via the use of substances was to isolate socially and physically in an environment already described as prison-like [6] and restrictive of personal autonomy [6,19]. This isolation resulted in increased experiences of boredom. Participants therefore felt they were left with only two constrained choices: to engage in substance use with their peers or to further subject themselves to the unrelenting feeling of boredom that accompanies social and physical isolation.

Another capability that participants didn’t have access to is that of *control over one’s environment*, which pertains to the ability to participate politically, the ability to own possessions, and engage in meaningful employment opportunities. Participants described little in the way of political control, often depicting themselves as passive service users who were at the whim of policies, procedures, and structural factors outside of their control. Despite the desire for meaningful activities within their environment, participants were powerless and unable to adequately advocate for the provision of these types of services and support. Additionally, participants lamented their inability to achieve material satisfaction and described their poverty as a major barrier to engagement in meaningful activities within the community. This lack of autonomy and financial independence resulted in the experience of boredom and the consequential utilization of substances as a means of escaping this unpleasant experience. Participants described their substance use an escape from the otherwise undiluted experience of profound boredom. Faced with limited opportunities for meaningful engagement within their environments or society generally, many participants utilized substances to achieve a different state of mind as other, normative means of emotional and cognitive release was perceived as inaccessible. One means to reduce barriers to control over one’s environment may be the improvement of employment support services for those experiencing homelessness, which current services struggle to accomplish [54]. The majority of persons experiencing homelessness desire the opportunity for employment [54], but are unable participate due to issues with substance use, shelter policies, engagement in treatment for mental health supports, and stigma associated with criminal histories or homelessness itself [55].

### Research and practice implications

While more research is needed, our findings suggest that the provision of meaningful activities for persons who are unhoused may have important implications regarding the experience of boredom and corresponding substance use. Recent literature has similarly suggested that addressing the issue of boredom within this demographic may also help address or alleviate the symptoms of mental illness and substance use in this population [5]. The positive influence of meaningful activities on reducing substance use has been identified both within unhoused populations [29] and the general population [56,57], yet these types of opportunities were perceived as unavailable by participants within this sample. This suggests that persons who are unhoused face a chronic deficiency in personal autonomy and are unable to pursue meaningful activities, supporting the notion that the experience of homelessness and boredom are concomitant [2,3]. The experience of boredom among persons who are unhoused may not appear important given the other issues that afflict individuals who experience homelessness, such as high rates of mental illness [48], morbidity, and mortality among persons who are unhoused [1]. However, addressing boredom may be an important step in reducing substance use by increasing meaning and purpose in their lives.

Future research should explore how the ability to engage in meaningful activities may impact the relationship between boredom and substance use within this population. This exploration may yield important outcomes insofar as it has the potential to reduce substance use in this demographic. A reduction in substance use may improve the mental health of a population of persons who already face high rates of mental illness [1,48]. Researchers should further explore the relationship between substance use, boredom and rumination on trauma or grief. This is especially true given the high prevalence of trauma among persons who are unhoused [58,59]. It may be promising to explore interventions that not only increase opportunities for meaningful activity but also support adaptive coping when individuals experience boredom or unstructured time. Approaches such as trauma-informed cognitive-behavioral strategies, mindfulness-based interventions, or emotion regulation training may help reduce the tendency to ruminate on distressing experiences and, in turn, lower the amount of substance use as a means of coping with unpleasant cognitions. Another promising avenue for future research is to explore how trait boredom, or the individual propensity to experience boredom regardless of environmental conditions, is related to the use of substances. Persons who are unhoused are more likely to experience boredom due to the higher prevalence of mental illness [1,48] and substance use [24] than the general population [5]. The increased tendency to experience boredom may itself serve as an additional barrier to attaining mental well-being for persons who experience homelessness. Further research is needed to determine if there are differences regarding the perceived relationship between boredom and substance use between persons who are using emergency shelters and those experiencing absolute homelessness (“street homelessness”). Research should also be conducted to examine the effects of addressing boredom as it relates to substance use and housing outcomes in transitional housing programs. Larger quantitative studies that focus on exploring the relationship between boredom and substance use within this population are also required.

Service providers should account for the importance of meaningful activities when supporting persons experiencing homelessness. There were many potential meaningful activities mentioned by participants in this study, though virtually all of them were unattainable due to environmental constrictions or socioeconomic marginalization. This inability to engage in meaningful activities despite the apparent desire to do so illustrates the inseparability of poverty, homelessness, and the experience of boredom. Previous research has shown that poverty negatively influences the ability to engage in meaningful leisure activities [34]. Within emergency and transitional housing environments, efforts should be made to ensure that free and equitable access to meaningful social and recreational opportunities are available to service users. Similarly, it is important that persons who are unhoused have access to free or low-barrier social and recreational opportunities within their respective communities thereby providing a greater degree of autonomy to be able to engage independently in meaningful activities.

### Policy recommendations

Policymakers may consider implementing approaches that mitigate the disadvantage that persons experiencing homeless face regarding access to meaningful activities such as evidence-based models of income support like universal basic income. Improving the financial situation of individuals experiencing homelessness will provide enhanced opportunities for accessing social and recreational opportunities [52]. Providing persons experiencing homelessness with the ability to engage in paid work may also be an important avenue for increasing individual autonomy. It is vital to ensure that persons who are working while unhoused are not financially penalized for doing so [34]. Considerations should also be made regarding the allocation of funding to specialized vocational programs with sufficient accessibility and flexibility to assist persons experiencing homelessness in securing and maintaining gainful employment. Given the poor efficacy of most existing employment support programs for those experiencing homelessness in terms of employment, mental well-being, housing tenure, community integration and substance use, novel or modified approaches are likely needed [60]. Additionally, policy makers should consider the development of accessible, low- or no-cost community engagement initiatives for individuals experiencing homelessness such as outdoor fitness installations or community-based workshops. These efforts should be accompanied by targeted outreach strategies and transportation supports to enhance participation.

### Limitations

The data used for this secondary analysis was derived from interviews with individuals experiencing homelessness in three predominantly Caucasian mid-sized urban communities in Canada and all participants identified as a binary gender (50% of them identified as men, the other 50% as women). The majority of participants in this study (77.8%) identified as Caucasian. As a result, the findings of this study should be transferred with caution to racialized persons. The reader should be aware that a person’s experiences of homelessness may differ from the findings of this study, particularly for persons who live in rural communities, or have non-binary gender identities. While the sample size (18 participants) allowed for rich descriptive texts to be identified, further research using quantitative methods with larger samples is needed to generate generalizable findings. Additionally, participants in this study were categorized as “homeless” and treated homogeneously.

It is also important to acknowledge that boredom represents only one aspect of a broader constellation of intersecting challenges that persons experiencing homelessness face. Because the original study was not designed to explore health-related factors such as trauma, physical illness, or mental health conditions, the relative influence of boredom on substance use should be interpreted with caution. The intent of this analysis was not to minimize these acute and deleterious issues but rather to highlight boredom as an underexamined, yet meaningful, contributor that interacts with other determinants to shape substance use behaviors.

## Conclusion

The findings of this analysis suggest that the experience of prolonged boredom among persons who are unhoused influences the propensity to engage in substance use. According to the participants in this study, engaging in substance use serves to mitigate the experience of boredom in a variety of ways including the facilitation of interpersonal relationships, coping with negative cognitions or trauma, managing unoccupied time, or providing stimulation otherwise unavailable within the environments in which they were situated. Enhancing opportunities to engage in meaningful activities needs to be facilitated through a range of policy and practice interventions. These may include implementing universal basic income programs to remove barriers to participation, the provision of meaningful activities within emergency shelter environments, and specialized vocational programs that can assist persons experiencing homeless to acquire and maintain gainful employment. Future research should endeavor to determine how the provision of meaningful activities addresses the prevalence of boredom among people who are unhoused and influences the relationship between boredom and substance use among persons who experience homelessness, and specific interventions that may be effective for facilitating engagement. Further research should also be directed at developing and evaluating effective ways to reduce barriers to engaging in meaningful activities for persons experiencing homelessness. The ability to engage in meaningful activities may have far reaching effects by reducing the high rates of substance use and improving the well-being of this population.
